# Comment on “The Relationship Between Ambulatory Arterial Stiffness Index and Incident Atrial Fibrillation”

**DOI:** 10.1002/clc.24333

**Published:** 2024-08-06

**Authors:** Mustafa Candemir, Emrullah Kızıltunç

**Affiliations:** ^1^ Department of Cardiology, Faculty of Medicine Gazi University Ankara Turkey

We read with great interest this observational cohort study with a median duration of 4 years by Boos et al. [1]. In this study, ambulatory arterial stiffness index (AASI) was found to be an independent predictor of the development of AF [1]. First of all, we would like to congratulate the authors of this article for raising awareness that parameters (such as AASI) obtained from ambulatory blood pressure monitoring (ABPM) have independent predictive value in many important diseases [2, 3]. We thought some points should be clarified so we decided to add some helpful comments on this article.

It is known that the diagnosis duration of patients with diseases like hypertension, heart failure, and diabetes may affect AASI, which provides information about arterial stiffness [3, 4]. Therefore, was there a statistically significant difference between the diagnosis duration of these diseases (hypertension, heart failure, and diabetes) in the AF and non‐AF groups?

In addition, the incidence of heart failure and ischemic stroke was higher in the AF group in the study. We know that these diseases have an impact on AASI [2]. Therefore, we think that it would be appropriate to include these diseases as confounding variables in the Cox regression analysis. The authors said that they limited the number of variables included in the regression model because the AF incidence was 9.1% (*n* = 75). However, in regression analysis, the number of events per variable can be between 5–9. It is known that the results of this analysis are correct [5]. Therefore, the number of variables evaluated in the regression analysis could have been increased to eight. Finally, the difference in β‐blocker use rates between groups may have caused AASI to lead to a statistically significant difference between the groups. Purifying the study results from the effects of the drugs used would also enable better interpretation of the results.

Despite these comments, we agree that this study will contribute greatly to the literature.

## Conflicts of Interest

The authors declare no conflicts of interest.

## Data Availability

The data that support the findings of this study are available on request from the corresponding author. The data are not publicly available due to privacy or ethical restrictions.
